# Effects of reduced rebreathing time, in spontaneously breathing patients, on respiratory effort and accuracy in cardiac output measurement when using a partial carbon dioxide rebreathing technique: a prospective observational study

**DOI:** 10.1186/cc3801

**Published:** 2005-09-07

**Authors:** Kazuya Tachibana, Hideaki Imanaka, Muneyuki Takeuchi, Tomoyo Nishida, Yuji Takauchi, Masaji Nishimura

**Affiliations:** 1 Staff physician, Surgical Intensive Care Unit, National Cardiovascular Center, Osaka, Japan; 2 Director, Surgical Intensive Care Unit, National Cardiovascular Center, Osaka, Japan; 3 Staff physician, Surgical Intensive Care Unit, National Cardiovascular Center, Osaka, Japan; 4 Staff physician, Surgical Intensive Care Unit, National Cardiovascular Center, Osaka, Japan; 5 Staff physician, Surgical Intensive Care Unit, National Cardiovascular Center, Osaka, Japan; 6 Professor, Department of Emergency and Critical Care Medicine, Tokushima University School of Medicine, Tokushima, Japan

## Abstract

**Introduction:**

New technology using partial carbon dioxide rebreathing has been developed to measure cardiac output. Because rebreathing increases respiratory effort, we investigated whether a newly developed system with 35 s rebreathing causes a lesser increase in respiratory effort under partial ventilatory support than does the conventional system with 50 s rebreathing. We also investigated whether the shorter rebreathing period affects the accuracy of cardiac output measurement.

**Method:**

Once a total of 13 consecutive post-cardiac-surgery patients had recovered spontaneous breathing under pressure support ventilation, we applied a partial carbon dioxide rebreathing technique with rebreathing of 35 s and 50 s in a random order. We measured minute ventilation, and arterial and mixed venous carbon dioxide tension at the end of the normal breathing period and at the end of the rebreathing periods. We then measured cardiac output using the partial carbon dioxide rebreathing technique with the two rebreathing periods and using thermodilution.

**Results:**

With both rebreathing systems, minute ventilation increased during rebreathing, as did arterial and mixed venous carbon dioxide tensions. The increases in minute ventilation and arterial carbon dioxide tension were less with 35 s rebreathing than with 50 s rebreathing. The cardiac output measures with both systems correlated acceptably with values obtained with thermodilution.

**Conclusion:**

When patients breathe spontaneously the partial carbon dioxide rebreathing technique increases minute ventilation and arterial carbon dioxide tension, but the effect is less with a shorter rebreathing period. The 35 s rebreathing period yielded cardiac output measurements similar in accuracy to those with 50 s rebreathing.

## Introduction

A partial carbon dioxide rebreathing technique has been developed to estimate cardiac output (CO) in mechanically ventilated patients undergoing surgery [1,2] or intensive care [3,4]. We previously reported that 50 s carbon dioxide rebreathing resulted in increased minute ventilation (V_E_) and an irregular respiratory pattern [4]. Recently, an improved system with a shorter rebreathing time (35 s) was developed and is replacing the 50 s rebreathing system. We reasoned that shortening the carbon dioxide rebreathing period would lessen the increases in arterial carbon dioxide tension (PaCO_2_) and respiratory effort during carbon dioxide rebreathing. We were concerned, however, that measurement of CO might be compromised by a shorter rebreathing period because there would be smaller changes in the measured variables, fewer sampled breaths and incomplete equilibrium [5]. We designed the present prospective study to investigate how, in spontaneously breathing patients, the shorter carbon dioxide rebreathing period affects respiratory effort during rebreathing and how it affects the accuracy of CO measurement.

## Materials and methods

The study was approved by the ethics committee of the National Cardiovascular Center (Osaka, Japan), and written informed consent was obtained from each patient.

### Patients

Thirteen consecutive patients (age 39–79 years) who had undergone elective cardiovascular surgery were enrolled in the study (Table 1). Enrolment criteria were similar to those of previous studies [3,4]: insertion of a pulmonary artery catheter, stable haemodynamics in the intensive care unit (ICU) and no leakage around the endotracheal tube. We excluded those patients who had central nervous system disorders, who might be adversely affected by induced hypercapnia, or who exhibited severe tricuspid regurgitation. After admission to the ICU each patient was ventilated with an 8400*STi *ventilator (Bird Corp., Palm Springs, CA, USA). Initial ventilatory settings were synchronized intermittent mandatory ventilation plus pressure support ventilation (PSV), volume controlled ventilation, tidal volume (V_T_) 10 ml/kg, respiratory rate 10 breaths/min, inspiratory time 1.0 s, positive end-expiratory pressure 4 cmH_2_O, and PSV 10 cmH_2_O. The inspired fraction of oxygen was adjusted by attending physicians to maintain arterial oxygen tension greater than 100 mmHg. Using an inspiratory hold technique, we measured the effective static compliance and resistance of the respiratory system (Table 1) [6]. In all patients, arterial blood pressure, heart rate, pulmonary artery pressure, central venous pressure and pulse oximeter signal (PM-1000; Nellcor Inc., Hayward, CA, USA) were continuously monitored. Patients were sedated with propofol (2–3 mg/kg per hour). After waiting 1–2 hours for haemodynamics to stabilize, we decreased the dosage of propofol to 1–2 mg/kg per hour.

### Study protocol

**Figure 1:**
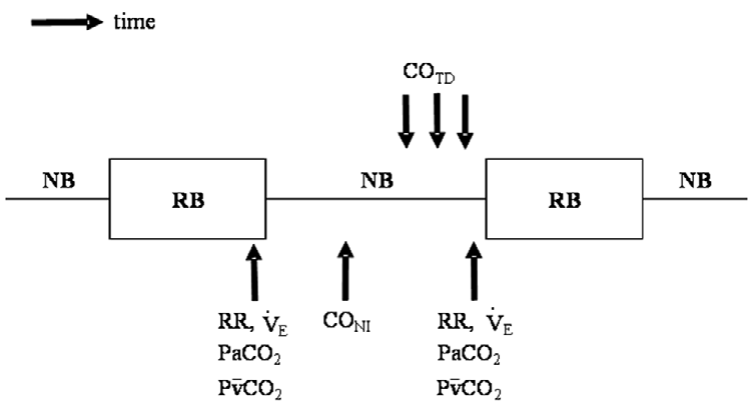
Schedule of measurements. Respiratory rate (RR), minute ventilation (V_E_), arterial carbon dioxide tension (PaCO_2_) and mixed venous carbon dioxide tension (PvCO_2_) were recorded both at the end of the normal breathing period (NB) and at the end of the partial rebreathing period (RB). At the middle of normal breathing period cardiac output using partial carbon dioxide rebreathing technique (CO_NI_) was measured; then, cardiac output using thermodilution technique (CO_TD_) was measured in triplicate and the values were averaged.

As each patient recovered spontaneous breathing, we gradually decreased synchronized intermittent mandatory ventilation rates, finally changing the ventilatory mode to continuous positive airway pressure with PSV at 10 cmH_2_O. The measurement protocol was started when the recruited patients satisfied the following conditions: recovery of cough reflex; V_T _≥ 8 ml/kg and respiratory rate ≤ 20 breaths/min; arterial blood gas of pH 7.35–7.45; PaCO_2 _35–45 mmHg; and arterial oxygen tension ≥ 100 mmHg at an inspired fraction of oxygen ≤ 0.5. We applied two systems of noninvasive partial carbon dioxide rebreathing technique in a random order. After waiting for at least 15 min, we recorded respiratory and haemodynamic data. Because the stimuli of partial carbon dioxide rebreathing increased spontaneous breathing, we recorded the data as displayed on the graphic monitors of the ventilators for respiratory rate and V_E _at the end of the normal breathing period and at the end of the rebreathing periods (Fig. 1). At the same times arterial blood was drawn via radial artery cannulation and mixed venous blood via pulmonary artery catheter; samples were analyzed with a calibrated blood gas analyzer (ABL 505; Radiometer, Copenhagen, Denmark).

### Cardiac output measurements

We randomly applied two systems of noninvasive partial carbon dioxide rebreathing technique to measure CO (CO_NI_): 35 s rebreathing (version 4.5, fast mode; Novametrix Medical Systems Inc., Wallingford, CT, USA) and 50 s rebreathing (version 4.2, fast mode). Although the durations of carbon dioxide rebreathing were different, both the total cycle (3 min) and the calculation algorithm were the same. Sensors for noninvasive partial carbon dioxide rebreathing technique (NICO_2_) were placed between the tracheal tube and Y-piece. The principle underlying this technique is described in detail elsewhere [3-5]. Briefly, carbon dioxide production (VCO_2_) is calculated on a breath-by-breath basis and a differential Fick equation is applied to establish the relationship between VCO_2 _and CO as follows:

VCO_2 _= CO × (CvCO_2 _– CaCO_2_)     (1)

Where CvCO_2 _is the carbon dioxide content in mixed venous blood, and CaCO_2 _is the carbon dioxide content in arterial blood. Assuming that both CO and CvCO_2 _remains constant during carbon dioxide rebreathing and that the change in CaCO_2 _between normal breathing and carbon dioxide rebreathing is proportional to the changes in PaCO_2 _and end-tidal carbon dioxide pressure (PETCO_2_), the following equation is substituted for the previous one:

CO = ΔVCO_2_/(S × ΔPETCO_2_)     (2)

Where ΔVCO_2 _is the change in VCO_2 _and ΔPETCO_2 _is the change in PETCO_2 _between normal breathing and carbon dioxide rebreathing, and S is the slope of the carbon dioxide dissociation curve from haemoglobin. After compensating, from the pulse oximeter signal, for the intrapulmonary shunt fraction, the partial carbon dioxide rebreathing technique obtains values for CO.

After we had acquired CO_NI _data, we measured thermodilution CO (CO_TD_) via a 7.5-Fr pulmonary artery catheter (Abbott Laboratories, North Chicago, IL, USA; Fig. 1). During the latter half of the normal breathing period, injection of 10 ml cold saline (0°C) was done three times and the values obtained were averaged. We carefully standardized the timing of bolus injections to after the first half of the expiratory phase [7].

### Statistical analysis

Data are presented as mean ± standard deviation, or as the median and interquartile range if the data were skewed. Comparison of respiratory rate, V_E_, PaCO_2 _and mixed venous partial carbon dioxide tension (PCO_2_) between different conditions (35 s versus 50 s rebreathing, and normal breathing versus rebreathing) were conducted using the Friedman test and the Wilcoxon signed rank test. We evaluated the agreement among CO_NI _with 35 s rebreathing, CO_NI _with 50 s rebreathing and CO_TD _using Bland-Altman analysis [8]. *P *< 0.05 was considered statistically significant.

## Results

### Respiratory loads

**Figure 2:**
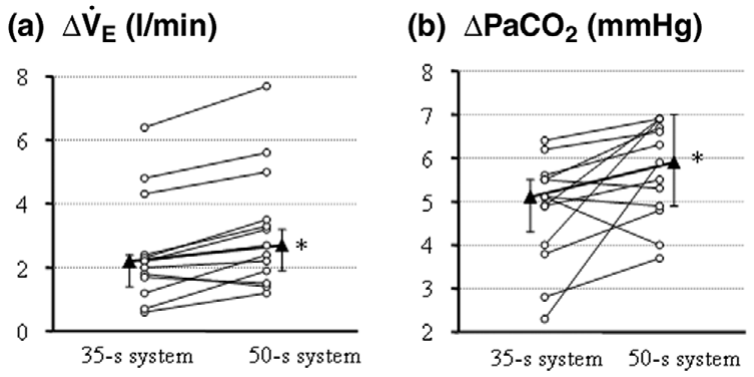
Changes in respiratory values in each patient due to carbon dioxide rebreathing. **(a) **Increases in minute ventilation (V_E_) due to carbon dioxide rebreathing. **(b) **Increases in arterial carbon dioxide tension (PaCO_2_) due to carbon dioxide rebreathing. Medians (triangles) and interquartile ranges are also shown. *P < 0.05 versus 35 s rebreathing.

Respiratory and blood gas results are summarized in Table 2. There was no significant difference in respiratory rate, V_E_, PaCO_2 _and mixed venous PCO_2 _during normal breathing between 35 s rebreathing and 50 s rebreathing (Table 2). With either duration of rebreathing, respiratory rate and V_E _increased during rebreathing. Similarly, the values for PaCO_2 _and mixed venous PCO_2 _were higher at the end of the rebreathing period. The changes in V_E _and PaCO_2 _due to rebreathing were significantly less with 35 s rebreathing than with 50 s rebreathing (Fig. 2).

### Cardiac output

**Figure 3:**
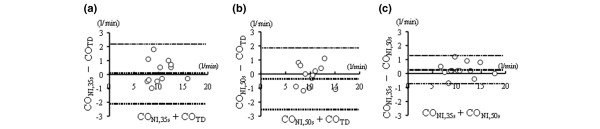
Bias analysis between cardiac output measurements. **(a) **Cardiac output obtained by partial carbon dioxide rebreathing of duration 35 s (CO_NI,35s_) and thermodilution technique (CO_TD_). **(b) **Cardiac output obtained by partial carbon dioxide rebreathing of duration 50 s (CO_NI,50s_) and CO_TD_. **(c) **CO_NI,35s _and CO_NI,50s_. Dotted lines show bias and limits of agreement between the two methods.

The results of Bland-Altman analysis for 35 s and 50 s rebreathing systems are summarized in Fig. 3. The CO measured using both systems exhibited similar agreement (bias and precision, respectively: 0.02 l/min and 1.06 l/min with 35 s rebreathing, and -0.34 l/min and 1.08 l/min with 50 s rebreathing) with values measured by thermodilution. When comparing the CO between 35 s rebreathing and 50 s rebreathing, bias was 0.26 l/min and precision was 0.51 l/min (Fig. 3c).

## Discussion

The main findings of the present study, conducted in spontaneously breathing patients, are that respiratory rate, V_E_, PaCO_2 _and mixed venous PCO_2 _increased during the rebreathing period; that increases in V_E _and PaCO_2 _during carbon dioxide rebreathing were less with the shorter rebreathing period; and that the two systems, with different rebreathing periods, provided similarly accurate CO measurements.

The NICO_2 _system is appealing as a noninvasive method for measuring CO in patients in whom pulmonary artery catheterization is not possible or desirable. Because it is now common for ICU patients to receive partial ventilatory support that allows spontaneous breathing [9], we must determine how the reduction in carbon dioxide rebreathing time affects respiratory effort and how accurate the NICO_2 _system is in such patients.

### Respiratory effort

One disadvantage of the partial carbon dioxide rebreathing technique is that rebreathing increases the respiratory effort of spontaneously breathing patients [4]. Consequently, the effect on respiratory effort of different durations of carbon dioxide rebreathing requires clarification. To our knowledge, no other investigations into this issue have been published. First, we found that the increase in PaCO_2 _during 50 s rebreathing was 5.9 mmHg (median; Fig. 2). These increases were greater than values (2–5 mmHg) previously reported in applications of controlled mechanical ventilation [10,11]. We assume that the greater metabolic rate in awake and spontaneously breathing patients accounted for the higher increase in PaCO_2 _during carbon dioxide rebreathing. Next, as we had conjectured, the shorter period of carbon dioxide rebreathing resulted in lesser increases in PaCO_2 _and, as a result, reduced the increases in V_E _during carbon dioxide rebreathing (Fig. 2). Although NICO_2 _monitoring is relatively noninvasive under controlled mechanical ventilation, it increases PaCO_2 _and respiratory effort under partial ventilatory support, even during 35 s rebreathing.

### Accuracy of cardiac output measurement

Although we previously found this technique to be less accurate when there were spontaneous breathing efforts [4], in the present study CO_NI _correlated moderately well with CO_TD_. We reason that we were able to obtain more stable V_T _and V_E _findings during CO measurement in the present study by using a larger dosage of propofol (1–2 mg/kg per hour) than in the previous study (0.5 mg/kg per hour). It is likely that stable V_T _and V_E _resulted in more accurate CO measurement. Gama de Abreu and coworkers [12], using a system different from ours, also reported that results were less precise when there was irregular spontaneous breathing than when respiratory rate and V_T _were fixed.

Because of smaller changes in the measured variables, fewer sampled breaths and incomplete equilibrium, we expected that the shorter duration of rebreathing would lead to less accurate CO measurement [5]. However, CO measurement with 35 s rebreathing was as accurate as with 50 s rebreathing (Fig. 3). Although the exact reason is unknown, we speculate as follows; Because the CO_NI _value is calculated from the ratio of change in VCO_2 _and PETCO_2 _during carbon dioxide rebreathing, the measurement is corrupted by noise and by variations in V_T _and respiratory rate [5]. Smaller carbon dioxide stimuli during 35 s rebreathing probably result in a more stable ventilatory pattern, whereas the smaller changes in VCO_2 _and PETCO_2 _during 35 s rebreathing lead to a poorer signal-to-noise ratio. In the range of durations tested, these two factors might proportionally cancel each other out, resulting in similar accuracy between 35 s rebreathing and 50 s rebreathing.

### Limitations

The present study has several limitations. First, we waited for 15 min after applying each NICO_2 _system with 35 s and 50 s rebreathing. When spontaneous breathing effort is present and V_E _is changing, more time may be required to attain stable conditions and an accurate CO_NI_. The time course of the increase in PaCO_2 _after a decrease in V_E _is much slower than the rate of decrease after an increase in V_E _[13]. Second, all of the patients included were sedated, but different levels of sedation may result in different responses to carbon dioxide rebreathing. Third, although the patients enrolled in this study exhibited normal lung mechanics (Table 1), critically ill patients with metabolic acidosis may respond differently to carbon dioxide rebreathing [14]. Although we speculate that our findings may be expanded to other patients with stable haemodynamics, and normal lung mechanics and gas exchange, further studies are needed to evaluate the accuracy and reproducibility of the NICO_2 _system with various levels of sedation and various patient populations. Fourth, the sample size in the study was small and we did not conduct a power analysis to determine the needed sample size. Because we performed multiple measurements in the same individuals, the order of measurements might have affected the results. Finally, the NICO_2 _algorithm assumes that mixed venous PCO_2 _remains constant during partial carbon dioxide rebreathing [5]. However, we found that increases in mixed venous PCO_2 _were larger than those previously reported (Table 2) [15,16]. When mixed venous PCO_2 _increases during carbon dioxide rebreathing, this must lead to an underestimation in CO_NI _[5]. Further study is needed to clarify the effects of the change in mixed venous PCO_2 _on the accuracy of CO measurement.

## Conclusion

When patients breathe spontaneously, CO measurement using partial carbon dioxide rebreathing technique increases PaCO_2 _and V_E_, although shortening the carbon dioxide rebreathing period causes a lesser increase. The two durations of rebreathing result in similar accuracy in measuring CO.

## Key messages

• 	The NICO_2 _monitor is claimed to measure CO noninvasively using the partial carbon dioxide rebreathing technique.

• 	When there are spontaneous breaths, partial carbon dioxide rebreathing increases V_E _and PaCO_2_.

• 	Use of a shorter duration of rebreathing (35 s versus 50 s) has smaller effects on respiratory effort in spontaneously breathing patients.

• 	The shorter duration of carbon dioxide rebreathing system yields a CO measurement that is similar in accuracy to that obtained with the previously used, longer duration of rebreathing.

## Abbreviations

CO = cardiac output; ICU = intensive care unit; NICO_2 _= noninvasive partial CO_2 _rebreathing technique; PaCO_2 _= arterial carbon dioxide tension; PCO_2 _= partial carbon dioxide tension; PETCO_2 _= end-tidal carbon dioxide tension; PSV = pressure support ventilation; VCO_2 _= carbon dioxide production; V_E _= minute ventilation; V_T _= tidal volume.

## Competing interests

The authors declare that they have no competing interests.

## Authors' contributions

KT designed the study, collected and analyzed the clinical data, and drafted the manuscript. HI designed the study, carried out data collection and analysis, and extensively revised the manuscript. MT designed the study and performed the statistical analysis. TN and YT participated in the analysis and interpretation of data. MN designed the study and extensively revised the manuscript. All authors read and approved the final manuscript.

## Figures and Tables

**Table 1:** Patient profile at study enrolment

Characteristic/parameter	Value
Number of patients	13
Male/female	8/5
Age (years)	64 ± 12
Height (cm)	160 ± 11
Body weight (kg)	58 ± 14
Operative time (min)	252 ± 50
Intraoperative dose of fentanyl (μg/kg)	21 ± 8
Carbon dioxide production (ml/min per kg)	2.6 ± 0.2
Dead space fraction	0.48 ± 0.02
Venous admixture fraction	0.08 ± 0.02
CO with thermodilution (l/min)	5.3 ± 2.1
Compliance of the respiratory system (ml/cmH_2_O)	49.8 ± 14.8
Resistance of the respiratory system (cmH_2_O·s per l)	12.0 ± 2.9
Background disease	
Coronary artery disease	6
Acquired valve disease	6
Thoracic aortic aneurysm	1

Values are expressed as mean ± standard deviation. CO, cardiac output.

**Table 2:** Respiratory parameters and blood gas analysis at normal breathing and rebreathing

Respiratory and blood gas parameters	35 s system	50 s system
Respiratory rate (breaths/min)		
Normal breathing	16 (15–18)	17 (15–17)
Rebreathing	18* (16–22)	19* (16–22)
Minute ventilation (l/min)		
Normal breathing	6.6 (5.9–7.4)	6.3 (6.2–7.3)
Rebreathing	8.8* (8.0–11.6)	9.5* (8.2–12.4)
Arterial carbon dioxide tension (mmHg)		
Normal breathing	42.1 (41.0–46.9)	42.2 (39.6–48.6)
Rebreathing	46.5* (43.5–52.5)	47.2* (45.9–55.0)
Mixed venous carbon dioxide tension (mmHg)		
Normal breathing	46.2 (44.4–52.2)	48.0 (43.9–52.2)
Rebreathing	47.6* (46.1–52.9)	49.0* (47.0–54.4)

Values are expressed as median (interquartile range). *P < 0.05 versus normal breathing.
